# Post-consultation acute respiratory tract infection recovery: a latent class-informed analysis of individual patient data

**DOI:** 10.3399/BJGP.2022.0229

**Published:** 2023-02-21

**Authors:** Hilda Hounkpatin, Beth Stuart, Shihua Zhu, Guiqing Yao, Michael Moore, Christin Löffler, Paul Little, Timothy Kenealy, David Gillespie, Nick A Francis, Jennifer Bostock, Taeko Becque, Bruce Arroll, Attila Altiner, Pablo Alonso-Coello, Alastair D Hay

**Affiliations:** School of Primary Care, Population Sciences and Medical Education, Faculty of Medicine, University of Southampton, Southampton, UK.; School of Primary Care, Population Sciences and Medical Education, Faculty of Medicine, University of Southampton, Southampton, UK.; School of Primary Care, Population Sciences and Medical Education, Faculty of Medicine, University of Southampton, Southampton, UK.; Department of Health Science, University of Leicester, Leicester, UK.; School of Primary Care, Population Sciences and Medical Education, Faculty of Medicine, University of Southampton, Southampton, UK.; Institute of General Practice, Rostock University Medical Center, Rostock, Germany.; School of Primary Care, Population Sciences and Medical Education, Faculty of Medicine, University of Southampton, Southampton, UK.; Department of General Practice and Primary Health Care, University of Auckland, Auckland, New Zealand.; Centre for Trials Research, College of Biomedical & Life Sciences, School of Medicine, Cardiff University, Cardiff, UK; Nuffield Department of Primary Care Health Sciences, University of Oxford, Radcliffe Observatory Quarter, Oxford, UK.; School of Primary Care, Population Sciences and Medical Education, Faculty of Medicine, University of Southampton, Southampton, UK.; Division of Health and Social Care Research, King’s College London, London, UK.; School of Primary Care, Population Sciences and Medical Education, Faculty of Medicine, University of Southampton, Southampton, UK.; Department of General Practice and Primary Health Care, University of Auckland, Auckland, New Zealand.; Department of Health Science, University of Leicester, Leicester, UK.; Iberoamerican Cochrane Centre, Instituto de Investigación Biomédica Sant Pau (IIB Sant Pau-CIBERESP), Barcelona, Spain.; Centre for Academic Primary Care, Population Health Sciences, Bristol Medical School, University of Bristol, Bristol, UK.

**Keywords:** antibiotics, general practice, latent class analysis, respiratory tract infections

## Abstract

**Background:**

There is a lack of evidence regarding post-consultation symptom trajectories for patients with respiratory tract infections (RTIs) and whether patient characteristics can be used to predict illness duration.

**Aim:**

To describe symptom trajectories in patients with RTIs, and assess baseline characteristics and adverse events associated with trajectories.

**Design and setting:**

The study included data about 9103 adults and children from 12 primary care studies.

**Method:**

A latent class-informed regression analysis of individual patient data from randomised controlled trials and observational cohort studies was undertaken. Post-consultation symptom trajectory (severity and duration), re-consultation with same or worsening illness, and admission to hospital were assessed.

**Results:**

In total, 90% of participants recovered from all symptoms by 28 days, regardless of antibiotic prescribing strategy (none, immediate, and delayed antibiotics). For studies of RTI with cough as a dominant symptom (*n* = 5314), four trajectories were identified: ‘rapid (6 days)’ (90% of participants recovered within 6 days) in 52.0%; ‘intermediate (10 days)’ (28.9%); ‘slow progressive improvement (27 days)’ (12.5%); and ‘slow improvement with initial high symptom burden (27 days)’ (6.6%). For cough, being aged 16–64 years (odds ratio [OR] 2.57, 95% confidence interval [CI] = 1.72 to 3.85 compared with <16 years), higher presenting illness baseline severity (OR 1.51, 95% CI = 1.12 to 2.03), presence of lung disease (OR 1.78, 95% CI = 1.44 to 2.21), and median and above illness duration before consultation (≥7 days) (OR 1.99, 95% CI = 1.68 to 2.37) were associated with slower recovery (>10 days) compared with faster recovery (≤10 days). Re-consultations and admissions to hospital for cough were higher in those with slower recovery (ORs: 2.15, 95% CI = 1.78 to 2.60 and 7.42, 95% CI = 3.49 to 15.78, respectively).

**Conclusion:**

Older patients presenting with more severe, longer pre-consultation symptoms and chronic lung disease should be advised they are more likely to experience longer post-consultation illness durations, and that recovery rates are similar with and without antibiotics.

## INTRODUCTION

Respiratory tract infections (RTIs) are one of the most common acute conditions managed in primary care and account for approximately 60% of antibiotic prescribing in primary care in the UK and continental Europe.^1^^–^^3^ Antibiotic prescribing remains widespread,^1^ despite studies showing antibiotic treatment has little effect on symptom duration.^4^^–^^7^

Studies have identified the importance of good clinician–patient communication in reducing inappropriate use of antibiotics.^8^^,^^9^ Patients often do not expect antibiotics for acute RTIs, but instead seek reassurance and information on what to expect.^10^^–^^12^ Similarly, physicians often face uncertainty about the natural course of RTIs and who is more likely to experience complications.^13^ There are relatively little data on the severity and duration of key symptoms for patients, and specifically adults.^14^^,^^15^ To the authors’ knowledge, only one study has previously described post-consultation symptom trajectories, and it was unable to identify baseline factors predictive of subsequent trajectory, other than prior severity of symptoms.^16^ A more complete understanding of the symptom trajectories associated with clinically identifiable patient and disease characteristics could guide patient assessment, help facilitate more personalised advice in consultations, and help reduce clinician uncertainty.^17^

This study aimed to describe patterns of change in severity and duration of symptoms in patients with acute and uncomplicated RTIs. Which baseline characteristics predict different trajectories of symptoms and whether adverse outcomes vary across trajectories was explored.

**Table:** How this fits in

There is a lack of evidence regarding post-presentation symptom trajectories for patients with respiratory tract infections (RTIs) and whether patient characteristics can be used to predict illness duration. For patients with RTI with cough, one in two patients will recover within 6 days of presentation, with the rest taking up to 1 month. Four trajectory groups were identified, two ‘faster’ and two ‘slower’; for clinical utility, these could be grouped into ‘faster’ and ‘slower’ recovery. Slower recovery was associated with older age, higher baseline severity, prior illness duration ≥7 days, and presence of lung disease. These characteristics can be easily assessed in primary care and could be used to provide more accurate prognostic information with patients and parents, and to manage expectations.

## METHOD

### Participants

Participants were from an individual patient data (IPD) database on antibiotic prescribing for RTIs (acute sore throat, acute cough, and otitis media) in a community setting (Box 1). Full details of the IPD database have been reported elsewhere.^18^^,^^19^ For the present study, participants in the IPD database who completed symptom diaries (*n* = 9103) were included. Study and patient-level characteristics are presented in Supplementary Table S1.^20^^–^^31^

### Diary data

For most studies, patients (or parents/caregivers of children aged <16 years) were asked to complete daily symptom diaries for the first 10 to 28 days (depending on the study) following their consultation or until symptoms returned to normal. Diary designs were based on previous validated formats.^24^^,^^32^ Symptom severity was rated from 0 to 6 (0 = no problem at all, 1 = very little problem, 2 = slight problem, 3 = moderate problem, 4 = bad problem, 5 = very bad problem, and 6 = as bad as can be). Symptoms recorded ranged across studies (depending on type of infection).

### Antibiotic group

Data on antibiotic consumption were available in some diaries (*n* = 4243). For diaries that did not collect this information, the authors assumed participants who were not prescribed antibiotics did not take antibiotics for the duration of the study period, and those who were prescribed immediate antibiotics were assumed to have taken antibiotics.^28^ Those prescribed delayed antibiotics were excluded from the longitudinal latent class analyses (LLCAs) described below.

### Baseline characteristics

Baseline data on sociodemographic variables included age (<16, 16–64, >64 years) and sex. Clinical variables were fever at baseline consultation (≥/<37.5ºC for each study), duration of illness before index consultation (≥/< the median), baseline severity of symptoms (average severity across all symptoms being ≥/< the median), presence of any lung disease (including asthma, chronic obstructive pulmonary disease, or any other lung disease).

### Outcomes

The primary outcomes of interest were symptom duration (both any symptom and individual symptoms) and change in symptom severity from index consultation. Duration of cough, sleep disturbance, feeling generally unwell, and interference with normal activities were examined as these were the symptoms for which data were collected in most studies. Change in severity of all symptoms collected in each study was examined. For incomplete diaries where the last observation was a value of 0 (‘no problem at all’), it was assumed symptoms had resolved on that day and a value of zero was assigned for all following days.

Secondary outcomes were re-consultation, defined as a consultation with the same or worsening illness (within 28 days following the index consultation) and admission to hospital.

**Box 1.** Individual patient data (IPD) database on antibiotic prescribing for respiratory tract infections (RTIs)

The IPD database included 13 studies (nine randomised controlled studies and four observational cohort studies) on antibiotic prescribing for RTIs (acute sore throat, acute cough, and otitis media) in a community setting. Studies were included in the IPD database if they compared delayed antibiotic prescribing with either immediate or no antibiotic prescribing. Studies were conducted in the UK, US, New Zealand, and 13 European countries, and included 55 682 participants. Studies were conducted between 1997 and 2016. Six of the 13 studies assessed all age groups, four studies assessed adult populations, and three studies focused on paediatric populations. Eleven studies were conducted in a primary care setting; one study was conducted in a paediatric emergency department; and one study was conducted in a paediatric clinic. Symptom severity data were not collected for all studies or were only collected for a subset of participants in some studies. Patients from studies that collected diary data were younger, more likely to have higher baseline severity of illness and shorter prior duration of illness, and were less likely to have any lung disease than patients in studies that did not collect diary data (see Supplementary Table S2).

### Data analysis

Study characteristics were described for all studies included in the analysis. Baseline characteristics of participants who provided symptom diary data were compared with those who did not. Data on the proportion of participants who were symptom free at each of the first 28 days post-consultation were plotted against time. Separate plots were produced for individual symptoms and for those who took and those who did not take antibiotics during the study period.

Trajectories of symptom severity (based on the most severe symptom on each day as previously defined)^14^^,^^28^ were modelled for participants. Separate trajectories were modelled for each initial diagnosis (that is, prevailing symptom at time of consultation). LLCAs were used to identify classes of participants with distinct trajectories of symptom severity^33^ using Mplus 8. LLCA is a type of mixture model that models patterns of states across time. Patients are assigned to classes/groups according to the probability of being in that class based on their symptom severity score at each time point (Box 2). Recovery time was defined for 90% rather than 50% of patients as this is thought to be more clinically useful and helpful to patients.

**Box 2.** Longitudinal latent class analysis (LLCA) method and assumptions

A series of models were fitted to determine the optimal number of classes. Model selection was based on the Bayesian Information Criterion, high entropy (≥0.80), good posterior probabilities (>0.8), and clinically relevant difference between classes. Full-information maximum likelihood estimation accounted for missing data, allowing the authors to use all available data and retain a larger sample size. For the LLCAs, three restrictions were made to reduce the data and allow model convergence:^16^ — data from the first 15 days post-consultation were included in the analyses; — data from alternate days (days 1, 3, 5, for example) were used to reduce the number of measures; and — symptom severity was re-grouped into three categories: 0, mild (severity scores 0–2); 1, moderate (scores 3–4); or 2, severe (scores 5–6). Symptom recovery time for each trajectory group was therefore described as time to symptom resolution to below moderate level.

Baseline characteristics of the different classes were compared using descriptive statistics (ANOVA for continuous variables and χ-square tests for categorical variables). Multinomial logistic regression models assessed which baseline characteristics predicted class membership, adjusting for confounders (age, sex, baseline severity, duration of illness before index consultation, fever at baseline, any lung disease, and antibiotic treatment group) and study ID variable. Multivariable logistic regression analysis assessed the association with class membership and outcomes of interest, accounting for clustering by study and confounders in observational studies. Multilevel multivariable logistic regression models assessed associations between recovery within 10 days (yes/no) with baseline characteristics and outcomes of interest. Analyses were repeated separately for adults (aged >18 years) and children.

## RESULTS

### Symptom duration

In total, 67.9% (*n* = 6179/9103), 24.4% (*n* = 2218/9103), and 7.8% (*n* = 706/9103) of participants had cough (as a dominant symptom), sore throat, and otitis media, respectively.

Data on time to symptom resolution were available for 11 studies (*n* = 8607). A flowchart showing inclusion and exclusion is available in Supplementary Figure S1. Median time to complete symptom resolution for these patients was 9 days (interquartile range [IQR] 4–27 days), with 90% of participants recovering within 28 days. Median time to complete symptom resolution was 15 (IQR 7–28) days for cough (90% recovered within 28 days), 4 (IQR 3–7) days for sore throat (90% recovered within 11 days), and 4 (IQR 2–8) days for otitis media studies (90% recovered within 10 days).

**Figure 1.**
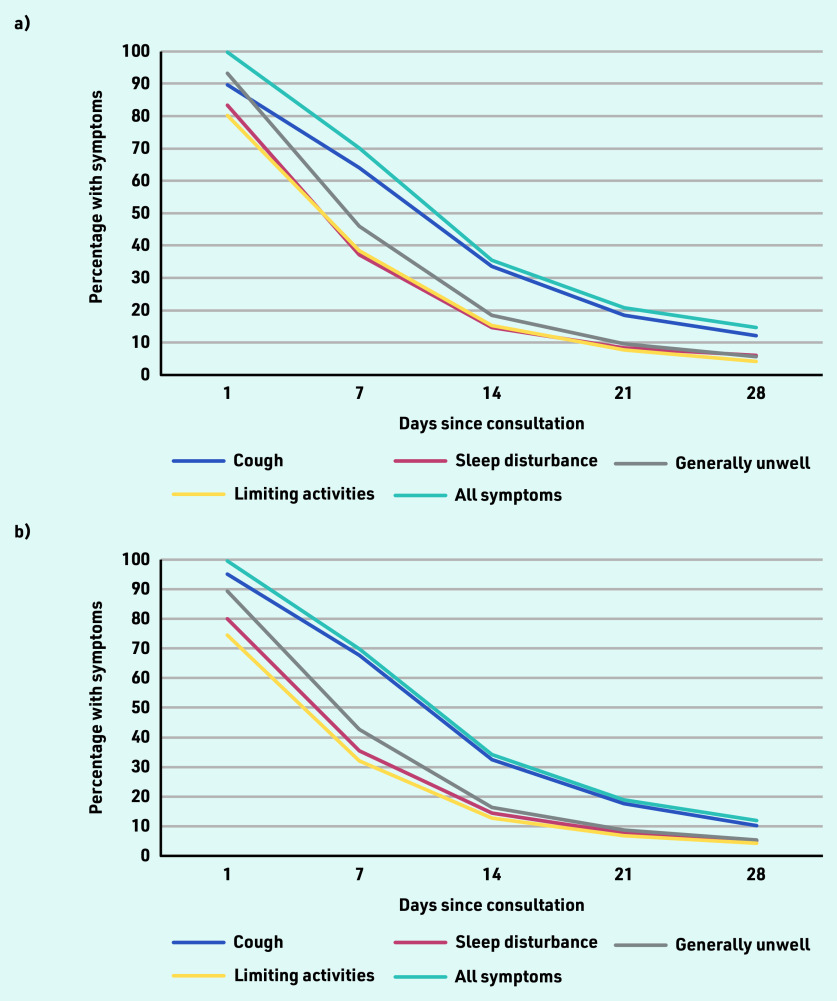
***Symptom resolution over the first 28 days after index consultation: a) for those who took antibiotics (*** **n *= 3637/7529); and b) for those who did not take antibiotics (*n *= 3892/7529).***

Participants who took antibiotics (48.3%; *n* = 3637/7529) had shorter median illness duration compared with those who did not take antibiotics (median: 9 [IQR 4–28] days versus 11 [IQR 5–28] days). However, 90% recovery was the same for those who took antibiotics and those who did not (28 days). A similar pattern was observed for those who took antibiotics during the study period (64.5% symptom free at 15 days; *n* = 2346/3637) and those who did not take antibiotics (65.9% symptom free at 15 days; *n* = 2565/3892). Over the first 28 days post-consultation, a larger percentage of people still had cough symptoms compared with other symptoms such as sleep disturbance, feeling generally unwell, and interference with normal activities (Figure 1).

Overall, the proportion of people who recovered within 1 week, 2 weeks, 3 weeks, and 4 weeks was 30.7% (*n* = 2067/6724), 65.8% (*n* = 3627/5509), 80.5% (*n* = 4451/5529), and 87.2% (*n* = 4335/4974), respectively. For adults, the proportion of people who recovered within 1 week, 2 weeks, 3 weeks, and 4 weeks was 26.1% (*n* = 1199/4594), 58.8% (*n* = 2043/3473), 76.5% (*n* = 2655/3470), and 84.4% (*n* = 2540/3008), respectively. For children, the proportion of people who recovered within 1 week, 2 weeks, 3 weeks, and 4 weeks was 40.8% (*n* = 868/2130), 77.8% (*n* = 1584/2036), 87.2% (*n* = 1796/2059), and 91.3% (*n* = 1795/1966), respectively. Among those without any lung disease, the proportion of people who recovered within 1 week, 2 weeks, 3 weeks, and 4 weeks was 30.3% (*n* = 1382/4556), 64.1% (*n* = 2254/3515), 79.6% (*n* = 2797/3514), and 86.9% (*n* = 2712/3121), respectively. Among those with lung disease, the proportion of people who recovered within 1 week, 2 weeks, 3 weeks, and 4 weeks was 25.6% (*n* = 220/861), 56.2% (*n* = 367/653), 69.7% (*n* = 456/654), and 76.8% (*n* = 468/609), respectively (data not shown).

### Symptom trajectories

Data on symptom severity were available for 6436 participants (from seven studies). Three of these studies focused on children and four on a general population. Five of the seven studies were on cough (*n* = 5314),^24^^,^^26^^,^^28^^,^^29^^,^^31^ one was on sore throat (*n* = 914),^27^ and one was on otitis media (*n* = 208).^23^

### LLCA trajectories

**Figure 2.**
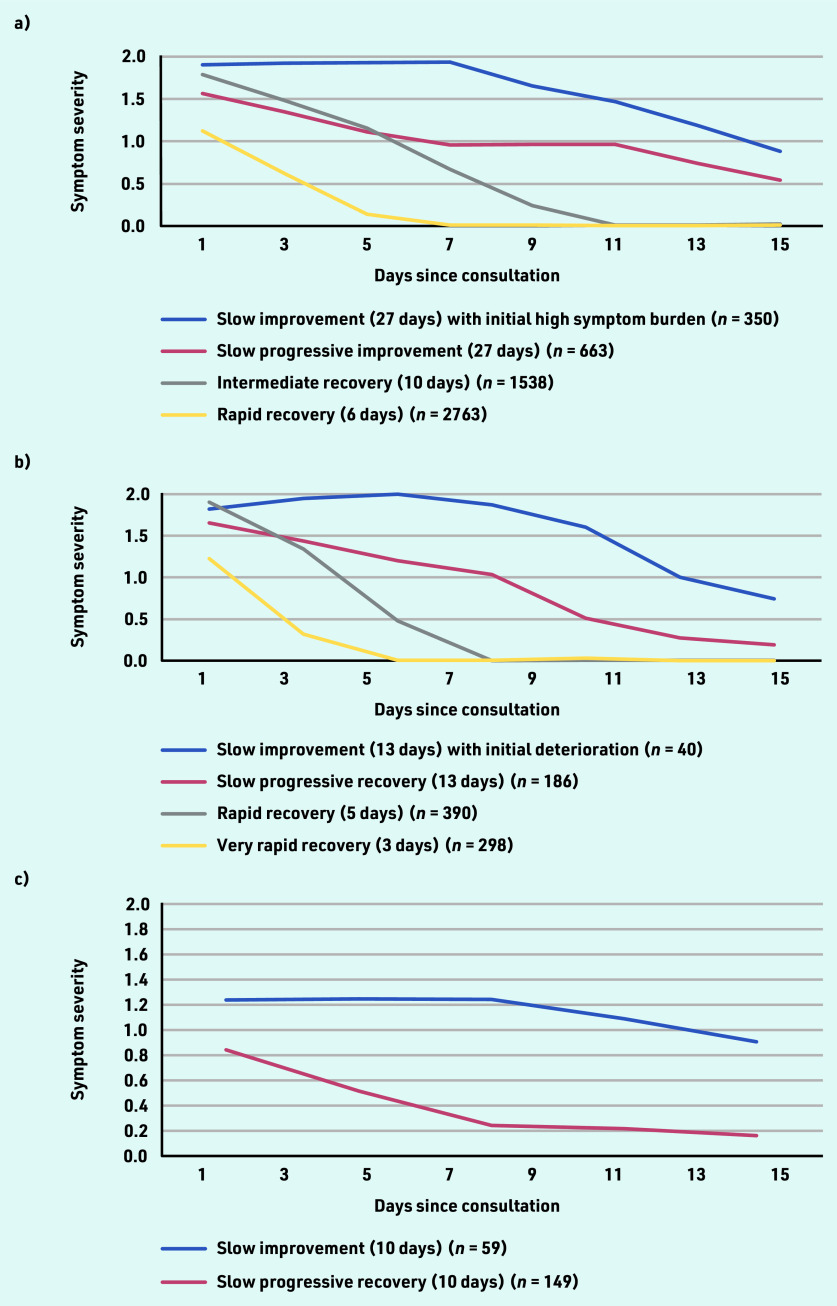
*Symptom trajectories based on longitudinal latent class analyses for a) cough; b) sore throat; and c) acute otitis media.*

LLCA for cough identified four trajectories with distinct patterns of change in symptom severity over time (see Supplementary Table S3). These trajectories and their distribution within the population were: ‘rapid recovery’ (52.0%; *n* = 2763/5314), ‘intermediate recovery’ (28.9%; *n* = 1538/5314), ‘slow progressive improvement’ (12.5%; *n* = 663/5314), and ‘slow improvement with initial high symptom burden’ (6.6%; *n* = 350/5314) (Figure 2). Time to symptom resolution to below moderate level for 90% of participants was 6 days for the ‘rapid recovery’ trajectory, 10 days for the ‘intermediate recovery’ trajectory, and 27 days for the ‘slow progressive improvement’ and ‘slow improvement with initial high symptom burden’ trajectories.

Baseline characteristics of the symptom trajectories for cough are presented in Table 1. Participants with ‘rapid recovery’ were generally younger and had slightly shorter duration of illness before the index consultation than participants in the remaining trajectory groups. A higher proportion of participants with ‘rapid recovery’ trajectory had lower baseline severity compared with the remaining trajectories. A lower proportion of participants with ‘rapid recovery’ were female or had any lung disease compared with the remaining trajectories. Similar results were obtained for adults and children.

**Table 1.** Baseline characteristics of all participants (adults and children) according to symptom trajectories for cough studies, *n*= 5314^a^

**Characteristic**	**Rapid recovery, 6 days (*n* = 2763, 52.0%)**	**Intermediate recovery, 10 days (*n* = 1538, 28.9%)**	**Slow progressive improvement, 27 days (*n* = 663, 12.5%)**	**Slow improvement with initial high symptom burden, 27 days (*n* = 350, 6.6%)**	***P*-value**
**Age, years, mean (SD)**	25.3 (24.4)	36.4 (22.8)	39.6 (24.2)	39.3 (22.7)	<0.001

**Sex, female, *n* (%)^b^**	1361 (54.3)	820 (60.9)	349 (60.1)	212 (67.5)	<0.001

**Baseline severity score, mean (SD)**	1.5 (0.6)	1.8 (0.7)	1.5 (0.6)	1.8 (0.6)	<0.001

**Prior duration illness, days, mean (SD)**	8.2 (7.0)	9.1 (7.2)	10.5 (7.3)	8.7 (5.9)	0.025

**Fever at baseline, *n* (%)^b^**	1269 (46.2)	745 (48.7)	293 (44.6)	152 (43.7)	0.163

**Any lung disease, *n* (%)^b^**	263 (14.2)	218 (17.1)	115 (21.6)	63 (21.1)	<0.001

**Treatment group, *n* (%)**					0.021
No antibiotics	1321 (47.8)	683 (44.4)	329 (49.6)	164 (46.9)	
Immediate	1031 (37.3)	647 (42.1)	260 (39.2)	136 (38.9)	
Delayed	411 (14.9)	208 (13.5)	74 (11.2)	50 (14.3)	

a P*-value <0.05 indicates statistically significant difference across groups. Data on sex and mean prior duration of illness were based on four studies.^26^^,^^28^^,^^29^^,^^31^*

b *Out of the 5314 participants, there were data available on fever at baseline for 5282 participants, data for any lung disease for 3957 participants, and data for sex for 4747 participants.*

*SD = standard deviation.*

Similar, although faster, trajectories were observed for sore throat and otitis media (see Supplementary Appendix S1).

### Faster versus slower recovery

To increase clinical usefulness, individuals were re-grouped into two groups each for cough and sore throat: ‘faster recovery (symptom recovery to below moderate levels within 10 days)’ and ‘slower recovery (symptom recovery to below moderate levels within 10 days)’.

#### Associations with baseline characteristics for cough

Older age (16–64 and >64 compared with <16 years) was associated with higher odds of slower recovery (odds ratios [OR] 2.57, 95% confidence interval [CI] = 1.72 to 3.85 and 3.17, 95% CI = 2.05 to 4.90, respectively). Median and above baseline severity was associated with ‘slower recovery’ compared with ‘faster recovery’: (OR 1.51, 95% CI = 1.12 to 2.03). Presence of lung disease (OR 1.78, 95% CI = 1.44 to 2.21) was also associated with ‘slower recovery’ compared with ‘faster recovery’. Median and above prior duration of illness (that is, ≥7 days) (OR 1.99, 95% CI = 1.68 to 2.37) were also associated with ‘slower recovery’ compared with ‘faster recovery’. Compared with no antibiotic prescribing, immediate antibiotic prescribing was associated with lower odds of slower recovery (OR 0.82, 95% CI = 0.68 to 0.98) (Table 2).

**Table 2.** Logistic regression analysis determining associations with slower (>10 days) versus faster recovery (≤10 days) for cough^a^

**Category**	**Cough, slower recovery (>10 days), OR (95% CI) (*n* = 1172, 20.3%)**
**Age, years**	
<16 (reference)	1.00
16–64	**2.57 (1.72 to 3.85)**
>64	**3.17 (2.05 to 4.90)**

**Sex, female**	1.05 (0.88 to 1.25)

**Baseline severity**	
Below median	1.00
Median and above	**1.51 (1.12 to 2.03)**

**Prior duration illness**	
Below median (<7 days)	1.00
Median and above (≥7 days)	**1.99 (1.68 to 2.37)**

**Fever at baseline**	
<37.5	1.00
>37.5	1.01 (0.84 to 1.20)

**Any lung disease**	
No	1.00
Yes	**1.78 (1.44 to 2.21)**

**Antibiotics**	
No antibiotics (reference)	1.00
Immediate	**0.82 (0.68 to 0.98)**
Delayed	0.99 (0.72 to 1.37)

a *Regression additionally adjusted for study ID variable as a random effect. OR = odds ratio. Bold indicates significance.*

#### Associations with re-consultation and admission to hospital. 

Re-consultation data were available for 5788 out of 6436 (89.9%) participants with symptom diary data (4714, 870, and 204 for cough, sore throat, and otitis media, respectively). Of these, 1685 (29.1%) re-consulted. Rates of re-consultation were 25.9% (*n* = 1015/3915) and 48.8% (*n* = 516/1057) in the ‘faster’ and ‘slower’ recovery group, respectively. Compared with the ‘faster recovery’ group, those in the ‘slower recovery’ group had increased odds of re-consultation (OR 2.15, 95% CI = 1.78 to 2.60).

Hospital admission data were available for 5915/6436 participants (91.9%). Hospital admission rates were 0.7% (*n* = 29/4021) and 2.5% (*n* = 27/1079) in the ‘faster’ and ‘slower’ recovery group, respectively. Compared with the ‘faster recovery’ group, those in the ‘slower recovery’ group had increased odds of admission to hospital (OR 7.42, 95% CI = 3.49 to 15.78).

Associations with the LLCA trajectories and baseline characteristics, re-consultation, and admission to hospital are available in Supplementary Tables S4– S7.

## DISCUSSION

### Summary

This study found that older age, greater baseline severity, longer prior duration of illness, and presence of lung disease predicted membership to trajectories with slower symptom recovery. Immediate antibiotic prescribing was associated with lower odds of slower recovery (that is, those prescribed immediate antibiotics were more likely to recover faster). Trajectories with slower recovery had higher odds of re-consultation and admission to hospital.

### Strengths and limitations

Strengths of the study include the large sample size, broad age group, and use of symptom diaries. The study brings together data from both observational cohort studies and randomised controlled trials, allowing greater confidence in the study findings.^34^

A limitation of the study was that not all studies collected data on symptom severity (resulting in 70.7% [*n* = 6436/9103] of participants being included in this analysis), although this is comparable with other studies.^16^ It is possible that those who did not provide data on symptom severity had different trajectories of change in severity and duration of symptoms. Similarly, patient confirmation of antibiotic consumption was available for 46.6% (*n* = 4243/9103) of participants. In the current study the authors assigned antibiotic group where possible based on prescribing strategy; however, some participants may have been misclassified, for example, if they obtained antibiotics through other sources or after the index consultation, and some people who were prescribed immediate antibiotics may not have taken them.^35^^,^^36^ However, evidence from trial data indicates that most people who are prescribed immediate antibiotics consume antibiotics.^28^

Missing data on symptom severity, antibiotic consumption, and outcomes resulted in a smaller sample size and limited statistical power for some of the analyses. The current study is consistent with previous studies that have reported little benefit with immediate antibiotics for RTIs. However, some associations may be confounded by patients in the slower recovery groups being more unwell or being more likely to have a bacterial infection. Additionally, self-reported symptom data may be prone to bias and individual differences in perception. Furthermore, in the current study the authors were not able to examine whether repeat antibiotic prescriptions influenced illness duration and trajectories as these data were not available in most studies. Similarly, the current study did not have data on previous admissions because of RTI. Furthermore, data were only analysed on symptom severity during the first 15 days following consultation. Although symptoms may last longer, most symptoms are resolved within the first 15 days as shown here and elsewhere.^16^ Finally, studies were conducted in high-income countries, and may not be generalisable to low- and middle-income countries or areas where there is higher prevalence of severe RTI (for example, tuberculosis).

### Comparison with existing literature

This study contributes to knowledge on the duration and severity of symptoms in patients with acute uncomplicated RTIs by describing trajectories of symptom recovery in a broad age group. The study findings are consistent with a previous study that identified five trajectories of cough severity and duration in children, which was predicted by prior severity of symptoms.^16^ The current study adds to these findings by identifying trajectories based on all symptoms and identifying patient characteristics associated with these trajectories. This present study extends this literature by illustrating slightly longer duration of symptoms (90% recovery time) and different patterns of symptom recovery in patients. This information is important given a recent study has suggested many patients are not aware of the natural history of RTIs and want to know when their symptoms are likely to improve.^37^ The current study of this general population also found longer duration of symptoms for cough, sore throat, and otitis media compared with that reported by a systematic review of symptom duration in children.^15^

### Implications for research and practice

A similar antibiotic prescribing rate was observed across the different groups for cough, highlighting clinician uncertainty and a need for a better guide about which patients should be prescribed antibiotics. Many key characteristics identified here such as illness severity, prior duration of illness, and presence of lung disease can easily be assessed in primary care and can be used by clinicians to reassure patients and appropriately manage those at risk.

Clinicians can advise patients with severe baseline severity, prior illness duration lasting >7 days, cough, or lung disease that they are likely to experience longer duration of symptoms (>10 days and up to 28 days) and should be alerted to the higher risk of admission to hospital.

This study found similar 90% recovery time and relatively small differences in odds of slower recovery for patients managed with and without antibiotics for cough. These findings support a ‘no’ or ‘watchful waiting’ approach to antibiotic prescribing and may help achieve current national targets to reduce antibiotic usage.^38^

The present study can be used to update the UK Health Security Agency guidance on RTI illness duration, which reports that most people will recover within 21 days for cough, 7–8 days for sore throat, 14 days for colds, and 8 days for otitis media.^39^ Characteristics of patients who are more likely to experience slower recovery or persistent symptoms may be additionally included in patient educational materials (for example, leaflets and videos) to help personalise prognosis.^39^ This could serve to modify patient expectations regarding illness duration and could reduce re-consultations.

Further research that more fully captures data on antibiotic consumption would allow more accurate estimates on the natural duration of symptoms. The current study may also inform risk prediction modelling, by highlighting which predictors may be included in models used to predict longer symptom duration or poorer outcomes for acute RTIs.

In conclusion, patients presenting with more severe and longer pre-consultation symptoms and chronic lung disease should be advised they are more likely to experience longer post-consultation illness durations, which can last up to 28 days, and that overall recovery rates are similar with and without antibiotics. This information can be included in educational resources that are provided to parents/patients, and patients with these characteristics should be alerted to the higher risk of admission to hospital.

## Data

The individual patient data database can be shared subject to the approval of the study collaborators.
